# Hands-on cybersecurity training behavior data for process mining

**DOI:** 10.1016/j.dib.2023.109956

**Published:** 2023-12-14

**Authors:** Radek Ošlejšek, Martin Macák, Karolína Dočkalová Burská

**Affiliations:** Faculty of Informatics, Masaryk University, Botanická 68a, Brno 60200, Czech Republic

**Keywords:** Puzzle-based gamification, Education, Learning analytics, Host-based data collection

## Abstract

The research on using process mining in learning analytics of cybersecurity exercises relies on datasets that reflect the real behavior of trainees. Although modern cyber ranges, in which training sessions are organized, can collect behavioral data in the form of event logs, the organization of such exercises is laborious. Moreover, the collected raw data has to be processed and transformed into a specific format required by process mining techniques. We present two datasets with slightly different characteristics. While the first exercise with 52 participants was not limited in time, the second supervised exercise with 42 trainees lasted two hours. Also, the cybersecurity tasks were slightly different. A total of 11757 events were collected. Of these, 3597 were training progress events, 5669 were Bash commands, and 2491 were Metasploit commands. Joint CSV files distilled from the raw event data can be used as input for existing process mining tools.

Specifications Table

**Table:** 

Subject	Computer Science Applications
Specific subject area	Practical exercises in cybersecurity: Tasks with trainees’ solutions in the form of event logs suitable for process mining.
Data format	Raw, Filtered, Aggregated/Normalized
Type of data	JSON, Table (CSV)
Data collection	We used the open-source KYPO Cyber Range [1] to set up hands-on training sessions and collect data automatically. This cyber range provides private sandboxes (emulated networks and hosts [2]) where practical cybersecurity tasks are performed. Also, it guides trainees through the exercise via a web interface. The usage of sandboxes was captured as command-line histories of individual trainees [3]. Their progress in the exercise, e.g., taking hints or advancing to the next task, was logged from the web interface. Collected raw data was processed and transformed into a format suitable for process mining techniques [4].
Data source location	Masaryk University, Brno, Czech Republic
Data accessibility	Repository name: Zenodo Data identification number: 10.5281/zenodo.10142981 Direct URL to data: https://zenodo.org/doi/10.5281/zenodo.10142981

## 1 Value of the Data

• The quality of hands-on cybersecurity training is crucial to mitigating cyber threats effectively. However, practical cybersecurity training is strongly process-oriented, making the application of learning analytics difficult. Process mining (PM) techniques [4] can help to analyze process-oriented data efficiently. Our data format fits the requirements put on the input of standard PM algorithms.
• Obtaining behavioral data through multiple approaches to the same cybersecurity task is laborious. Although the utilization of existing cyber ranges can help in this endeavor, the requirements on hardware and human resources remain enormous when collecting reasonably big representative data collections. We contribute to the scientific community by sharing the data we have collected.
• The data captures the real behavior of trainees participating in exercises mimicking realistic attack scenarios. As far as we know, this is the first complex data collection from this application domain directly usable in PM algorithms and PM-based analytical tools.
• The data collection is primarily intended for researchers in applied cybersecurity or cybersecurity education who aim to use PM techniques for behavioral analysis, e.g., to detect flows in training scenarios, cheating, or to identify attack-defense strategies of participants.
• As the data collection also includes the description of the training scenario (i.e., expected steps of trainees leading to the correct solution), it is possible to use the data for conformance analysis.
• Pre-processed PM-ready data and raw JSON data included in the collection can be used for direct non-PM analysis, e.g., to build hypotheses about trainees' behavior that are to be verified by PM models.

## 2 Background

While hands-on training, in general, produces a tangible output in many learning areas, e.g., a code that can be checked, analyzed, and evaluated, cybersecurity training is strongly process-oriented, producing only sparse behavioral logs. These logs are not directly usable for answering questions like “At which point did the trainees get in trouble and why?” Some analytical tools are needed to aggregate a more abstract insight into trainees’ behavior from this low-level data. Process mining represents an emerging discipline with the potential to answer these types of questions during post-training evaluation. However, PM poses some minimal requirements for the data. Also, the quality and practical usability of PM models for learning analytics are influenced by many factors. We aim to foster further research in this area by providing datasets that can be directly used in PM tools for the behavioral analysis of trainees. Thanks to the presence of expected behavior in our datasets, conformance analysis can be conducted along with process discovery.

## 3 Data Description

There are two distinct datasets in the collection [5] containing events from two training events. They share the same format and principles described in this section.

The **data1** dataset features event logs of an exercise involving 52 trainees. In total, 5270 events were recorded (1617 training events, 2749 Bash commands, and 904 Metasploit commands). On average, each trainee submitted 31 training events (min: 20, max: 46, median: 31), 52.8 Bash commands (min: 5, max: 188, median: 42), and 17.3 Metasploit commands (min: 0, max: 85, median: 12).

The **data2** dataset features event logs of an exercise involving 48 trainees. In total, 6487 events were recorded (1980 training events, 2920 Bash commands, and 1587 Metasploit commands). On average, each trainee submitted 41.25 training events (min: 25, max: 59, median: 41), 60.8 Bash commands (min: 7, max: 301, median: 46), and 33 Metasploit commands (min: 0, max: 180, median: 25).

### 3.1 Data structure

**Fig. 1:**
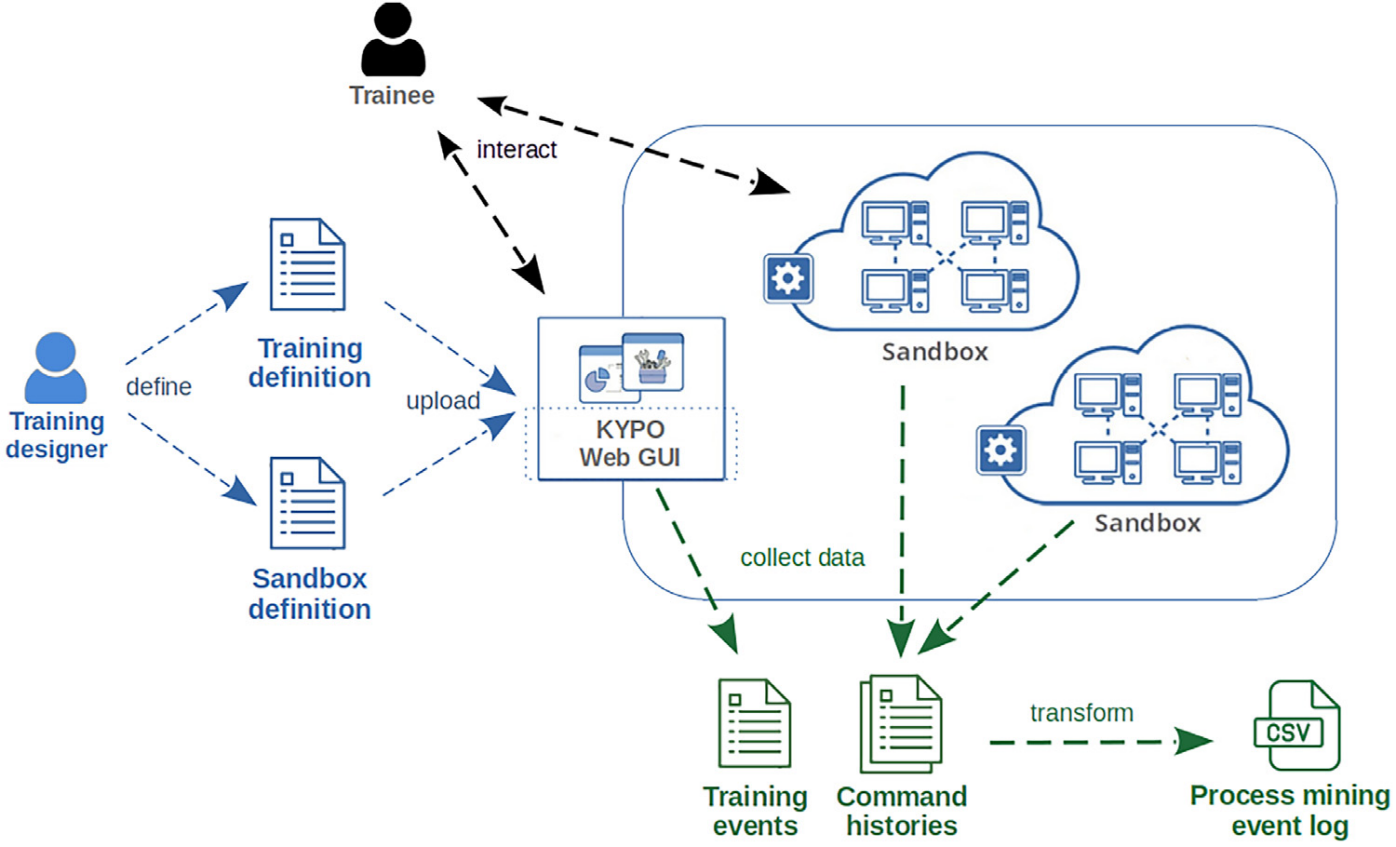
The KYPO CRP high-level architecture. The diagram outlines the training lifecycle, involved user roles and important data artifacts.

Files included in the data collection were either specified by content creators – training designers who prepare the exercise – or automatically generated by the KYPO Cyber Range in which the exercise is conducted. The complete life cycle of hands-on training with roles and data artifacts is schematically depicted in Fig. 1.

The content of an exercise is defined by two primary structured documents: (a) A *training definition* that describes the training scenario and (b) a *sandbox definition* capturing the topology of the sandbox, i.e., a virtual computer network in which the cybersecurity tasks are performed. These artifacts are prepared by training designers and used to set up a training session. They are included in our datasets to provide an overview of training content and expected behavior.

Every trainee has their own isolated copy of the sandbox. When the training session is open, trainees enter the training web portal, log into the computers of their sandbox, and follow instructions, aiming to solve prescribed cybersecurity tasks. Their activities are monitored. The web portal automatically logs trainees' progress in the form of *training events*. Simultaneously, activities in the sandbox are logged as *command histories*.

Once the training is completed, the collected events are exported and transformed into a *process-mining event log* suitable for process-mining analysis.

The datasets are organized with respect to data artifacts of the training life-cycle. The directory structure is captured in Table 1. In what follows, we describe the content of the relevant data files in detail.

**Table 1:** Directory structure of datasets.

file/directory	Description
training_definition.json	The exercise content – cybersecurity tasks and hints.
training_events/	Recorded progress of trainees within the exercise, i.e., the status of completing tasks.
command_histories/	Recorded commands executed on network hosts.
process_mining.csv	Complete PM-ready dataset suitable for process discovery or conformance analysis.
process_mining_simplified.csv	Reduced PM-ready dataset with semantically identical events being removed.

### 3.2 Training definition

The training definition is available in the “training_definition.json” file. It prescribes cybersecurity tasks that are displayed to trainees in the web portal during the session and can be used to repeat the training session in the KYPO Cyber Range to collect additional data.

The scenario is based on the Locust 3302^1^ [6] game. The topology of a computer network in which the cybersecurity tasks are performed consists of three hosts running Kali Linux. The employee host with IP 10.1.135.83 serves as the primary computer for the trainee from which the tasks are performed. The web server with IP 172.18.1.5 runs a vulnerable service. The client host has to be discovered by the trainee.

While the content of the **data2** dataset follows the Locust 3302 game, tasks to be solved by trainees in the **data1** dataset were simplified and adapted to address the insider attack scenario [7]:

• A trainee is put into a story where they are an employee of an IT company that deals with many important business data. Server administrator Eve was fired the previous week. However, she managed to change the credentials to the server with essential data before she left the company, leaving the server inaccessible. A trainee aims to get into that server and recover the company data stored there.
• To do that, the trainee has to use the employee computer with Kali Linux available in the sandbox to find a vulnerable service on the web server.
• Once the vulnerability is found, the trainee has to exploit it to get inside the server.
• The bad news is that Eve deleted the data from the server. However, she could make a copy so that she could extort money from the company later on. The goal is to find where the data was copied to.
• Fortunately, the server with the data copy is a server of our friendly client willing to provide us with the data for recovery.

Although the training definition file primarily aims to provide the wording of cybersecurity tasks, the file also includes many additional supporting data fields. In what follows, we focus on records that are important for understanding trainees' behavior and interpreting their behavioral data files. Omitted fields can be considered technical or related to assessment. As they don't appear in the final PM-ready CSV event log, they can be ignored.

The main section of the JSON file is the *levels* field that defines a sequence of cybersecurity tasks that are to be solved by trainees one by one. However, the levels are of two types. *INFO_LEVEL* only provides instructions to trainees. No behavioral data is collected about trainees in this level type. On the contrary, *TRAINING_LEVEL* represents a cybersecurity task and consists of the following key sub-fields:

• *content* provides wording of the task and then instructs a trainee about the expected next step in the training session.
• *solution* is shown to a trainee on his or her demand. It summarizes the steps leading to solving the task. Showing the solution by the trainee is usually penalized.
• *hints* are also shown only on the trainee's request and penalized. In contrast to the complete solution, hints provide only partial help at the different levels of detail.
• *answer* is a secret string, also called a flag, which has to be found by a trainee by correctly solving the task. The flag has to be typed by the trainee in the web portal to continue to the next level.
• *reference_solution* stores expected commands used by trainees on sandbox hosts. As these fields represent reference walkthroughs with optional and alternative traces, they can be used as a reference model for conformance process analysis [4].

### 3.3 Training events

Training events capture the interaction of individual trainees with the training web portal. This data traces the progress of trainees in the training session in terms of milestones (solving a task and proceeding to the next level) and hints, as shown in the snippet in Listing 1.

**Listing 1:**
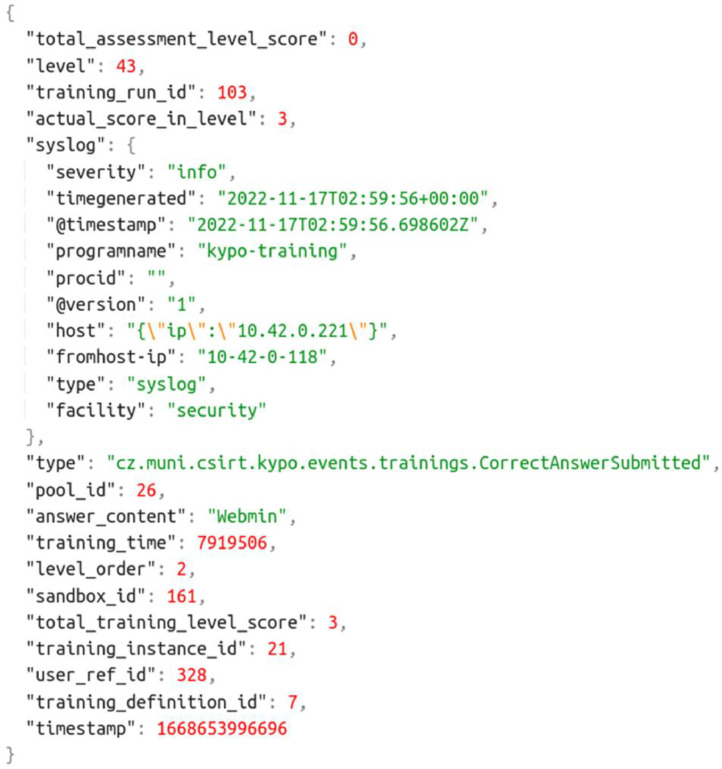
JSON structure of training events.

Training events are stored in files named “training_run-id*-events.json” under the “training_events” folder. For the PM-based reconstruction of the trainee's behavior, four pieces of information are crucial, while unmentioned fields can be ignored:

• Identification of a trainee is needed for distinguishing individual traces of behavior. The *user_ref_id* field serves directly as an identifier of the trainee who produced the training event. However, this field is not present in other data sources, especially files with sandbox command histories. Fortunately, trainees can be identified indirectly via the *sandbox_id* field, which is present in all files. This field uniquely identifies the sandbox of the user. And because each trainee has access to a single sandbox and vice versa, it can be used to uniquely identify users as well.
• The level at which the event occurred is needed for finding correspondence between cybersecurity tasks and behavioral data. Information about game levels is spread across multiple fields: the *level, level_order, level_type*, and optional *level_title*. The *level_order* is irrelevant for the reconstruction of real traces. For quick reference, Tables 2 and 3 summarize level IDs, their type, and titles that appear in data stored in the data1 and data2 datasets, respectively. Only the events of the *Training* type levels are relevant for behavioral analysis because they store activities reflecting the solving of cybersecurity tasks.

**Table 2:** Training levels appearing in the dataset1.

Level (ID)	Level type	Level title
62	Info	Questionnaire
42	Info	Introduction
43	Training	Check the remote services of the server
44	Training	Identify a vulnerability to get remote access
45	Training	Exploit the vulnerability and then get into the server
46	Training	Find a computer possibly storing a copy of the company data
49	Info	Congratulations

**Table 3:** Training levels appearing in the dataset2.

Level (ID)	Level type	Level title
78	Info	Prerequisite questionnaire
33	Info	Introduction to Locust 3302
34	Training	Scan the IP address
35	Training	Identify a vulnerability
36	Training	Exploit the vulnerability
37	Training	Find an IP address of the secret server
38	Training	Access the secret server
39	Info	Congratulations
40	Info	Post-training feedback

• *type* field provides the semantics of the activity. Recorded values are summarized in Table 4. Some events can be equipped with additional data, e.g., the value of the provided answer.

**Table 4:** The meaning of training events and their optional parameters.

Event type	Additional data field	Description: The trainee ...
TrainingRunStarted		… started the training.
TrainingRunEnded		… finished the training.
LevelStarted		… started a level.
LevelCompleted		… finished a level.
WrongAnswerSubmitted	answer_content, correct_answer	… submitted the wrong flag.
CorrectAnswerSubmitted	answer_content	… submitted the correct flag.
HintTaken	hint_title, hint_penalty	… took a hint.
SolutionDisplayed		… viewed the step-by-step solution of the task.

• *@timestamp* sub-field of the *syslog* record provides the time of the event's occurrence. The format fits the ISO 8601 format (up to millisecond or microsecond precision). Other time records listed in the game event log are irrelevant for process analysis.

### 3.4 Command histories

Sandbox events stored in files “sandbox-id*-useractions.json” under the “command_histories” folder capture commands used by trainees on sandbox hosts. An example of an event is shown in Listing 2.

**Listing 2:**
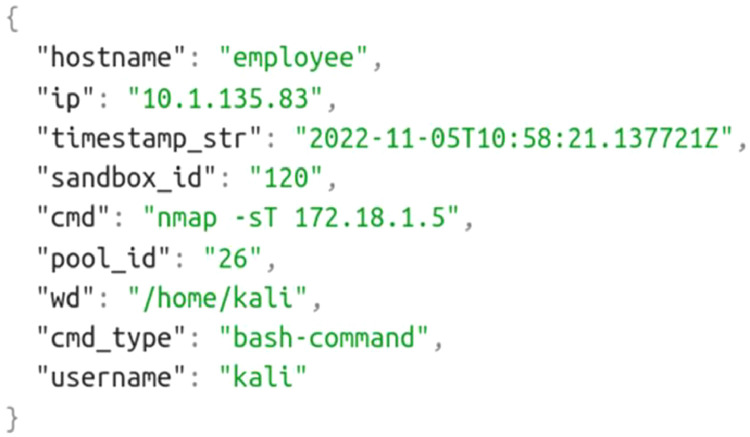
JSON structure of command history events.

The meaning of the individual data fields follows:

• *hostname* is the name of a host in the sandbox on which the command was executed. The name corresponds to some name defined in the “topology.yml” file.
• *ip* is the host's IPv4 address as defined in the “topology.yml” file.
• *cmd* is the command with parameters used by the trainee on the host.
• *cmd_type* classifies commands into categories. The value is either “bash-command” for Shell commands or “msf-command” for the Metasploit tool.
• *wd* is the working directory in which the Bash command was executed. This field is omitted for the “msf-command” command type.
• *timestamp_str* stores the command's execution time in the ISO 8601 format.
• *sandbox_id* uniquely identifies the sandbox in which the events were collected. This number also uniquely identifies a trainee because each trainee has access to a single sandbox and vice versa.
• *pool_id* is a technical field used by the KYPO Cyber Range Platform to maintain sandboxes during training sessions. This field is not used for process mining.
• *username* is the host's system account name under which the Bash command was executed. This field is not used for process mining.

### 3.5 Process mining event log

*Training events* and *command histories* collected by the KYPO Cyber Range were processed and merged into CSV files matching the input data requirements of process mining algorithms [4].

In what follows, we describe the structure and meaning of the obtained data fields shown in Listing 3:

• *sandbox_id* uniquely identifies the user regardless of whether the record is a training or sandbox event. This column can be used as a process mining case ID to reconstruct walkthroughs of individual trainees.
• *cmd_type* distinguishes Bash commands, Metasploit tool usage, and training events. The first two types (“bash-command” and “msf-command” values) are extracted from command histories, and the last one (the “event” value) is extracted from the training event logs. This column can be used for abstraction-driven events filtering [8] and then reducing the complexity of process graphs.
• *cmd_name* includes either the commands executed in sandboxes (without arguments) or training events. This column can be used as the main process mining activity.
• *cmd_arguments* field stores arguments of commands executed in sandboxes (without the command itself) or additional data provided to training events, e.g., a value of the correct or incorrect answer. The “NONE” keyword indicates events with no extra arguments. Possible joining selected parts of this field with the *cmd_name* value can affect the complexity and meaningfulness of obtained process graphs [7,15]. For example, joining the “ssh” *cmd_name* value with the remote address arguments such as “ssh 172.18.1.5” and “ssh 10.1.135.83” will force process mining algorithms to treat these commands as distinct.
• *hostname* column stores information about the source that produced the event. The value either corresponds to the name of a host in the sandbox or is set to the “KYPO Portal” value for training events.
• *ip* complements the *hostname* data with the information about the IP address of the event source. Therefore, the value either corresponds to the IPv4 address of the sandbox host or the IP address of the KYPO web portal.
• *level* is the ID of the training level in which the event appeared.
• *username* stores a user under which a Bash command was executed on the sandbox host. This value can be useful if the trainee can switch between identities in a single sandbox host. Nevertheless, for Metasploit commands, the value is unknown and then set to “null”. For training events, the value is set to the user ID.
• *timestamp_str* corresponds to the *timestamp_str* field of a sandbox event or *@timestamp* field of a training event. Therefore, this field stores the absolute time of the event's appearance. These values are recorded directly by the KYPO Cyber Range, but they are not very suitable for process mining. This is because the algorithms are very sensitive to the correct ordering of events. As the trainees never start the training exactly at the same time, the absolute time stamps do not reflect the correct ordering of events among multiple trainees.
• *relative_timestamp_str* was introduced to provide the correct ordering of events among multiple trainees. This column should be used as process mining timestamps to trace walkthroughs of multiple trainees correctly. The value represents a relative time computed as the difference between the absolute time recorded by the cyber range and the start of the trainee's activities in the training session.

**Listing 3:**
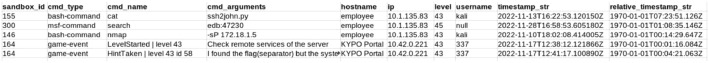
CSV structure of the process mining event log.

To open the CSV files in a spreadsheet editor, use commas as a separator. To use the files as input for process mining algorithms:

1. Choose the *relative_timestamp_str* column as the timestamps field.
2. Choose the *sandbox_id* column as the case field.
3. Choose the *cmd_name* column as the activity field.
4. Optionally filter lines by the *level, sandbox_id* (i.e., users), *cmd_type*, or other event properties to reduce the complexity of process graphs and pay attention to desired aspects of training.
5. Optionally extend selected *cmd_name* fields with values from *cmd_arguments* to subtly differentiate between commands.
6. For “bash-command” and “msf-command” *cmd_type* lines, optionally join *cmd_name* with the *level* column to differentiate between using the same command in solving different cybersecurity tasks.

## 4 Experimental Design, Materials and Methods

This section describes the steps conducted to get PM-ready datasets for process discovery and conformance analysis, including data gathering, aggregation, and cleansing. Also, the background of trainees involved in the measured cybersecurity hands-on exercise is discussed.

### 4.1 Participants

For the **data1** dataset, the online KYPO training environment was open for almost one month, from 5.11.2022 to 1.12.2022. Trainees could attend at any time, but each trainee could participate only once. It was enforced by providing a unique access code to them. The participants included undergraduate and graduate students of computer science from the Masaryk University in Brno, Czech Republic. During this time, 73 trainees attended the exercise, but only 52 finished all tasks.

For the **data2** dataset, the hands-on training was organized on 4.5.2022 as an on-site supervised exercise limited to two hours. 48 undergraduate and graduate students of computer science from the Masaryk University in Brno, Czech Republic, played the Locust 3302.

### 4.2 Data collection

The behavioral data of trainees was collected directly by the KYPO Cyber Range infrastructure and exported in JSON format. Then, the data was cleaned and processed to reflect the requirements put on the process mining data format.The raw JSON data is available in the dataset along with the processed PM-ready files.

### 4.3 Data aggregation and transformation

Because the KYPO Cyber Range platform uses two data sources, the web portal for game events and sandboxes for trainees' behavior on hosts, it produces two types of raw data files with different JSON structures. Therefore, all training events and sandbox events were merged into a single CSV file format of the *process mining event log*. During this process, the following aggregation and transformation steps were performed. All these transformations can be automatized and reproduced by using our simple Java application, which is distributed together with the datasets.

Relative time (the *relative_timestamp_str* column) was computed as the difference between the absolute time record and the start of the trainee's activities in the training session.

Because the same training event, e.g., “LevelStarted”, may appear in multiple training levels, all training events in the *cmd_name* column were extended with the level ID, e.g., “LevelStarted | level 46”. Otherwise, the obtained process models do not reflect reality. The “HintTaken” were, in addition, extended with hint ID for the same reason, e.g., “HintTaken | level 45 id 87”.

Bash and Metasploit commands are captured as a single text line in the raw JSON data. They were split into the command and argument parts by considering the first word as the command name and the remaining text as arguments. Similarly to training events, the same Bash or Metasploit command can also be used in multiple training levels. However, in this case, considering them the same or different commands strongly depends on the aims of the analyst. Therefore, we did not extend the commands with the level ID suffix by default. In general, it is supposed that analysts using our dataset will re-distribute the content of the *cmd_name* and *cmd_arguments* fields as part of their process-mining experiments [8,16].

Because Bash and Metasploit commands contain text written by a trainee, we sanitized the content by replacing end-of-line characters with the (newline) mark, commas with the (comma) mark, and quotes with the (quote) mark.

There are two versions of CSV files available in each dataset. The “process_mining.csv” file includes all events captured by the KYPO Cyber Range and process as discussed. However, some events can be considered duplicates, as they do not bring any useful semantics for process mining. Specifically, the *TrainingRunStarted* event is always followed by the *LevelStarted* event of the first training level, and the *LevelCompleted* is always followed by the next *LevelStarted* or the *TrainingRunEnded* events. Therefore, they are omitted in the “process_mining_simplified.csv” file.

### 4.4 Related work

Gamification of in-class hands-on training sessions represents a popular teaching approach [9], enabling tutors to collect and analyze data about trainees' behavior and then improve the impact of the learning on students. Data from serious games are used for learning analytics in many learning areas, including IT education [10,11].

The shortage of data suitable for learning analytics of practically oriented cybersecurity training sessions was caused by the lack of environments where such hands-on training could be easily organized and the data collected. It has changed in the past decade when virtualized cyber ranges appeared [12]. Since then, cyber ranges become an important source of data for evidence learning in cybersecurity exercises [13]. However, few datasets remain publicly available, consisting of raw shell commands, system logs, network traffic data, or event logs from cyber exercises.

Similar to datasets published in [14,15], our data was captured in the open-source KYPO Cyber Range [1], but they are unique in combining and synchronizing gamification-based training events with command histories. This type of process-oriented data seems to be useful for process-mining data analysis [8,16]. We believe that publishing this dataset will foster further research on using process discovery and conformance analysis techniques in learning analytics of cybersecurity exercises.

## Limitations

By analyzing training events of the **data1** dataset, we found 20 trainees who did not finish the whole training session. We identified them by missing data, especially evident by the missing “training completed” event, which is the last training record logged automatically by the KYPO Cyber Range. Training and sandbox JSON files of these participants were removed from the dataset. Moreover, we identified a few JSON records in the command histories that violated the expected structure of JSON files. These errors were introduced by the data-gathering infrastructure of the KYPO Cyber Range and were caused by unparseable texts written by users on command lines. These corrupted lines were deleted from raw JSON files.

The analysis of the **data2** dataset did not reveal any significant inconsistencies, and therefore, this dataset was kept unchanged.

## Ethics Statement

All data exported from the KYPO Cyber Range and used in the dataset are anonymized, avoiding revealing the identity of specific individuals. In general, the issues of collecting data in the KYPO Cyber Range were discussed with the ethical committee of our university. We obtained a waiver from the committee since we intentionally do not collect any personally identifiable information.

All participants were informed about the anonymized collection of the data for research purposes. They agreed via informed consent.

As command histories may include any text typed by trainees on a command line, we carefully checked the data, aiming to eliminate the risk of leaking some personal data in this way.

## CRediT authorship contribution statement

**Radek Ošlejšek:** Conceptualization, Methodology, Software, Data curation, Writing – original draft. **Martin Macák:** Conceptualization, Validation, Data curation, Investigation, Writing – original draft. **Karolína Dočkalová Burská:** Software, Resources, Formal analysis, Data curation.

## Data Availability

Dataset: Behavior of Participants in Hands-on Cybersecurity Training Suitable for Process Mining (Original data) (Zenodo)

## Footnotes

1 https://gitlab.ics.muni.cz/muni-kypo-trainings/games/locust-3302.
